# Fundamental Irreversibility: Planckian or Schrödinger–Newton?

**DOI:** 10.3390/e20070496

**Published:** 2018-06-27

**Authors:** Lajos Diósi

**Affiliations:** Wigner Research Centre for Physics, H-1525 Budapest 114, P.O. Box 49, Hungary; diosi.lajos@wigner.mta.hu

**Keywords:** fundamental irreversibility, space-time fluctuations, spontaneous state reduction

## Abstract

The concept of universal gravity-related irreversibility began in quantum cosmology. The ultimate reason for universal irreversibility is thought to come from black holes close to the Planck scale. Quantum state reductions, unrelated to gravity or relativity but related to measurement devices, are completely different instances of irreversibilities. However, an intricate relationship between Newton gravity and quantized matter might result in fundamental and spontaneous quantum state reduction—in the non-relativistic Schrödinger–Newton context. The above two concepts of fundamental irreversibility emerged and evolved with few or even no interactions. The purpose here is to draw a parallel between the two approaches first, and to ask rather than answer the question: can both the Planckian and the Schrödinger–Newton indeterminacies/irreversibilities be two faces of the same universe. A related personal note of the author’s 1986 meeting with Aharonov and Bohm is appended.

## 1. Introduction

Standard micro-dynamical equations, whether classical or quantum, are deterministic and reversible. They can, nonetheless, encode various options of irreversibility even at the fundamental level. Here, I am going to discuss two separate concepts of fundamental irreversibility, which are quite certain to overlap in the long run. The first option concerns space-time (gravity); it is relativistic, hallmarked by mainstream cosmologists and field theorists (including immortal ones). The second option is rooted in the explicit irreversibility of von Neumann measurement in non-relativistic quantum mechanics; its story is perhaps more diffusive than that of the first. The standard and linear story of Planck scale irreversibility is recapitulated in Section 2. I choose a personal account for the parallel story of the conjectured Newton-gravity-related non-relativistic irreversibility of macroscopic quantum mechanics in Section 3. I stop both stories in the 1980s when the same structure of heuristic master equations was proposed for the two options of fundamental irreversible dynamics—with different interpretations and regimes of significance, of course. Towards their reconciliation, Section 4 offers some thoughts and concludes in an open-ended fashion.

## 2. Irreversibility at Planck Scale

At the dawn of quantum-gravity research, Bronstein [1,2,3] discovered by heuristic calculations that the precise structure of space-time, contrary to the precise structure of electromagnetism, is unattainable if we rely on the quantized motion of test bodies. Subsequent decades raised stronger and famous arguments concerning space-time blurriness, unpredictability, its role in universal loss of information, of quantum coherence, and of microscopic reversibility in general. Wheeler [4] found that smooth space-time changes into a foamy structure of topological fluctuations at the Planck scale. Bekenstein [5] gave the first exact quantitative proposal toward fundamental irreversibility, claiming that black holes have entropy:

(1) $$S=\frac{k_{B}}{4\ell_{Pl}^{2}}\times \left(\mathrm{black}\;\mathrm{hole}\;\mathrm{surface}\;\mathrm{area}\right),$$

where $k_{B}$ is Boltzmann’s constant; $\ell_{Pl}$ is the Planck length. This was confirmed by Hawking [6] who showed that black holes do, indeed, emit the corresponding thermal radiation. A short time later, he summarized the situation by stating the unpredictability of quantum-gravity at the Planck scale, leading him to propose that quantum field theory is fundamentally irreversible [7]. Accordingly, the unitary scattering operator $\hat{S}$ should be replaced by the more general superscattering operator $ acting on the initial density operator ${\hat{\rho }}_{in}$ instead of the initial state vector:

(2) $${\hat{\rho }}_{out}=\${\hat{\rho }}_{in}\neq \hat{S}{\hat{\rho }}_{in}{\hat{S}}^{†}.$$

To resolve the detailed irreversible (non-unitary) dynamics beyond Hawking’s superscattering, Ellis et al. [8] proposed a simple quantum-kinetic (master) equation, which Banks, Susskind and Peskin [9] generalized as follows:

(3) $$\frac{d\hat{\rho }}{dt}=-\frac{i}{\hbar }\left[\hat{H},\hat{\rho }\right]-\frac{1}{2\hbar^{2}}\int \;\;\int \left[\hat{Q}\left(x\right),\left[\hat{Q}\left(y\right),\hat{\rho }\;\right]\right]h\left(x-y\right)d^{3}xd^{3}y,$$

where $\hat{H}$ is the Hamiltonian, $\hat{Q}\left(x\right)$ is a certain quantum field, and h(x−y) is a positive symmetric kernel. The transparent structure allowed the authors to point out a substantial difficulty: non-conservation of energy-momentum.

## 3. Irreversibility in the Schrödinger–Newton Context

In the early 1970s, being a student fascinated already by quantum theory, I missed a dynamical formalism of the state vector collapse from it. If I were a student and aware of the related literature, I would have read the phenomenological model by Bohm and Bub [10]. However, I was not aware of it, and started to think on my own. If you open a textbook, you will read about the expansion of the time-dependent state vector |t〉 in terms of the energy eigenstates |n〉 of eigenvalues $E_{n}$, respectively. However, I wrote it with a little modification:

(4) $$|t\rangle =\sum_{n}c_{n}\exp\left(-\frac{i}{\hbar }E_{n}\left(1+\delta \right)t\right)|n\rangle ,$$

because I observed that by allowing a small randomness δ of the time flow, the average density matrix becomes gradually diagonal in the energy basis:

(5) $$\bar{\left|t\rangle \langle t\right|}⟶\sum_{n}|c_{n}{|}^{2}|n\rangle \langle n|,$$

exactly as if someone measured the energy. I made a prototype dynamical model of non-selective von Neumann measurements. A question remained unanswered: where does randomness of time come from? The hint should have come from the sadly forgotten Bronstein [1,2,3], but it came from Károlyházy after he gave department seminars in 1973 on his earlier work [11] where he used a Planck scale uncertainty of classical space-time and a very vague model of massive body’s state vector collapse based upon it. Unfortunately, I had to do experimental particle physics for a decade.

Returning to theory, I showed [12] that the Newtonian limit of standard reversible semi-classical gravity, the so-called Schrödinger–Newton equation [13], obtains sensible solitonic wave functions for the massive (e.g., nano-) objects’ center-of-mass. This determined my approach, i.e., to put *non-relativistic* flesh on the toy dynamics (4) and (5) of state vector reduction. The uncertainty δ of time flow should come from the Newtonian limit of the metric tensor element $g_{00}$, which is, in fact, the Newton potential ϕ. The unpredictability δϕ of the Newton potential should depend on *G* and ℏ, but not on *c*. The choice was the following spatially correlated white-noise:

(6) $$\bar{\delta \phi (x,t)\delta \phi (y,s)}=\frac{\hbar G}{\left|x-y\right|}\delta \left(t-s\right).$$

The random part of the Newton potential couples to the mass density operator $\hat{f}\left(x\right)$ via the interaction $\int \phi \left(x,t\right)\hat{f}\left(x\right)d^{3}x$, yielding the following master equation for the density operator:

(7) $$\frac{d\hat{\rho }}{dt}=-\frac{i}{\hbar }\left[\hat{H},\hat{\rho }\right]-\frac{G}{2\hbar }\;\;\int \;\;\;\int \left[\hat{f}\left(x\right),\left[\hat{f}\left(y\right),\hat{\rho }\;\right]\right]\frac{1}{\left|x-y\right|}d^{3}xd^{3}y.$$

This dynamic is mimicking the (non-selective) von Neumann measurement of massive object’s positions. It predicts the *spontaneous reduction* (decay) of Schrödinger cat states (see the same result in [13] by Penrose).

Before journal publication [14], I showed this result to Yakir Aharonov (read Appendix A). He warned me about the energy-momentum non-conservation. This came as a surprise to me as I had not read [9].

## 4. Planck Scale or Schrödinger–Newton Context?

Irreversibility at the Planck scale seems plausible within standard physics because of evaporating black holes (Section 2). Non-relativistic Schrödinger–Newton irreversibility (Section 3) is a conjecture, although its derivation is not much more heuristic than that of Planckian’s. For both options, the same structure of master equations was proposed to encode the irreversible dynamics of the density operator. Planck scale irreversibilities from Equation (3) become significant for certain fundamental elementary particles. Contrary to that, Equation (7) predicts irreversibility for massive non-relativistic objects in the Schrödinger–Newton context. Whether the two underlying concepts are compatible at all is unknown. Whether the Newtonian unpredictabilities/fluctuations are the non-relativistic limit of the Planckian’s? That is difficult to answer.

Let me mention, nonetheless, two examples where relativistic phenomenologies, different from the line of Section 2, turned out to reduce to the Schrödinger–Newton uncertainty (6) non-relativistically. Unruh [15] proposed a possible uncertainty relation between the metric and Einstein tensors, respectively. In the Newtonian limit, speed of light *c* cancels out and we are left with just the white-noise uncertainties (6), as pointed out in [14]. Penrose discussed the fundamental conflict between general relativity and quantization. To resolve it, heuristically at least, he also found the necessity of space-time’s fundamental blurriness, guessed it non-relativistically and determined its equivalent with expression (6) up to a factor of 2 (a discrepancy which has recently been resolved by [16]).

Against questioning a possible transmutation of Planck scale uncertainties into the non-relativistic Schrödinger–Newton regime, I have an elementary argument. Consider the Schrödinger-equation for the center-of-mass of a big body such as *M* = 1 kg, with velocity 1 km/s which is fairly non-relativistic. Calculate the de Broglie wave length: $\lambda =\left(2\pi \hbar /mv\right)=4.16\times 10^{-36}$ m. This is smaller than the Planck length $\ell_{Pl}=1.62\times 10^{-35}$ m by about one order of magnitude. Since standard physics breaks down anyway at the Planck scale, we can no longer trust in the Schrödinger equation for the motion of our massive non-relativistic body. Planck scale space-time uncertainties have thus developed uncertainties in the Schrödinger dynamics of non-relativistic massive bodies. So far so good. However, will *c* be cancelled out so that we obtain the effective Schrödinger–Newton uncertainty (6) and (7) and the corresponding spontaneous reduction for massive objects [13,14]?

## 5. Concluding Remarks

Two independent theories of relativistic and non-relativistic fundamental irreversibility, both related to the conflict between gravity and quantization, are in the scope of this work. One was conceived and would be relevant in cosmology. The other one was born from the quantum measurement problem and would modify the quantum mechanics of massive bodies even in the lab. Their conceptions have been outlined in Section 2 and Section 3, respectively, including their basics without details and later developments. Such a restricted presentation sufficed to expose the issue at the center of this work in Section 4: what is the relationship between the Planckian and the Schrödinger–Newton unpredictability of our space-time? The question remains unanswered, but our purpose has been to highlight it. In particular, we pointed out that Planckian unpredictability survives non-relativistically—for massive macroscopic quantized degrees of freedom.

## Figures and Tables

**Figure A1:**
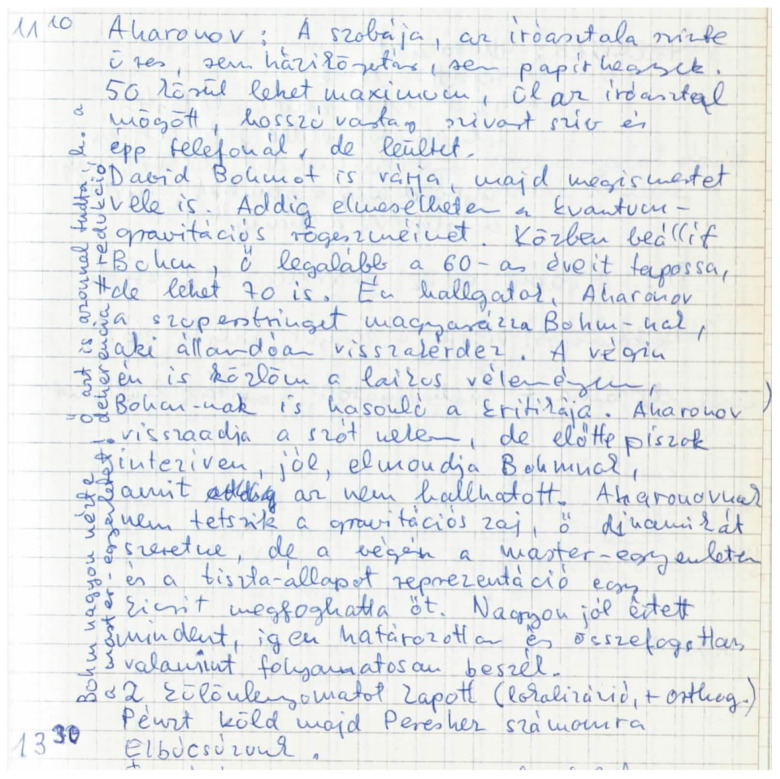
Author’s diary page, from 18 March 1986.

## Appendix A

It was Asher Peres who asked Yakir to receive the unknown theorist from Hungary. Below is the translation of my notes (Figure A1).
11^10^ Aharonov: His office and desk are almost empty, no personal library, no paper piles. He is at most 50 or so. He sits behind the desk, smokes a long fat cigar, makes a phone call, and asks that I take a seat. We await David Bohm, who I will also be introduced to. Until then, I can unfold my quantum-gravity idée fix. David Bohm arrives. He is at least in his 60s, but could be 70. I am listening as Aharonov explains the superstring to Bohm who is repeatedly asking questions. Finally, I also communicate my layman’s views; Bohm’s criticism is also akin. Aharonov allows me to speak, but first tells Bohm with hellish intensively what he could not have heard. Aharonov dislikes gravitational noise; he prefers dynamics. However, at the end, my master equation and the pure state representation may have caught him a bit. He understood everything very well, he spoke steadily, with real firmness and organization. He got two offprints (localization + orthog.) Peres will send money for me. 13^30^ We say goodbye. *Left margin:* Bohm looked at the master equation intently! Immediately, he also knew that decoherence ≠ reduction.
